# Role of widows in the heterosexual transmission of HIV in Manicaland, Zimbabwe, 1998–2003

**DOI:** 10.1136/sti.2008.033043

**Published:** 2009-03-13

**Authors:** B A Lopman, C Nyamukapa, T B Hallett, P Mushati, N Spark-du Preez, F Kurwa, M Wambe, S Gregson

**Affiliations:** 1 Imperial College London, London, UK; 2 Biomedical Research and Training Institute, Harare, Zimbabwe; 3 London School of Economics, London, UK; 4 Loughborough University, Loughborough, UK

## Abstract

**Background:**

AIDS is the main driver of young widowhood in southern Africa.

**Methods:**

The demographic characteristics of widows, their reported risk behaviours and the prevalence of HIV were examined by analysing a longitudinal population-based cohort of men and women aged 15–54 years in Manicaland, eastern Zimbabwe. The results from statistical analyses were used to construct a mathematical simulation model with the aim of estimating the contribution of widow behaviour to heterosexual HIV transmission.

**Results:**

413 (11.4%) sexually experienced women and 31 (1.2%) sexually experienced men were reported to be widowed at the time of follow-up. The prevalence of HIV was exceptionally high among both widows (61%) and widowers (male widows) (54%). Widows were more likely to have high rates of partner change and engage in a pattern of transactional sex than married women. Widowers took partners who were a median of 10 years younger than themselves. Mathematical model simulations of different scenarios of sexual behaviour of widows suggested that the sexual activity of widow(er)s may underlie 8–17% of new HIV infections over a 20-year period.

**Conclusions:**

This combined statistical analysis and model simulation suggest that widowhood plays an important role in the transmission of HIV in this rural Zimbabwean population. High-risk partnerships may be formed when widowed men and women reconnect to the sexual network.

AIDS as the leading cause of death among adults in southern Africa^1^ is the major driver of early widowhood, even in areas of low prevalence.^2^^–^6 In Zimbabwe an estimated 25% of men and women aged 15–49 years were infected with HIV in 2003,^7^ and the resulting mortality in these age groups means that children are orphaned and spouses are widowed, thus eroding family structures.^8^ ^9^ Owing to the relatively high levels of adult mortality even before the HIV epidemic, family structures were maintained by traditional intra- and intergenerational coping mechanisms such as the levirate, whereby a widow is remarried—often to a member of the deceased husband’s family.^10^^–^12 As the epidemic progresses and populations become aware of the impact of AIDS mortality, such practices may diminish.^13^ ^14^ Remarriage of widows involves people with a sexual history and therefore may account for some of the serodiscordant couples (where one person is HIV positive) observed in cohorts in sub-Saharan Africa.^3^ ^15^ Serodiscordant couples have a higher risk of widowhood than seroconcordant-negative couples, and seroconcordant-positive couples have the highest risk of widowhood, in which case an HIV-positive widow(er) is always produced.^16^

Previous findings from the Manicaland HIV/STD Prevention Study cohort and others in the southern African region, as well as modelling estimates, suggest that men mostly acquire HIV infection from premarital and extramarital relations while, for married women, the strongest determinants of infection are their partner’s behaviours.^9^ ^17^ ^18^ Marriage is a potentially risky form of partnership since sex occurs more frequently and condom use may be low.^19^ In Kisumu, Kenya and Ndola, Zambia, married women reported fewer partners than single women. However, husbands were 2–3 times more likely to be infected than boyfriends, thus cancelling out the protective effect of married women’s own behaviour. Clearly, marriage is only protective against sexually transmitted diseases (STDs) if it is monogamous and coincides with initiation of sexual activity.^20^ ^21^

Because the HIV epidemic first spread widely in men, because the background male mortality is normally higher because husbands are generally older than wives, and because men may take more than one wife, 78% of widows in Zimbabwe in 1999 were women.^22^ Importantly in terms of the further spread of HIV, the prevalence of HIV among widows is extraordinarily high. In the baseline round of the Manicaland HIV/STD Prevention Study cohort (1998–2000), men and women who were widowed <2 years before the study had the highest HIV prevalence of all marital status groups (64% and 58%, respectively).^9^ Studies in other Zimbabwean populations have also observed a similar risk of HIV in widows.^23^ ^24^

The demographic structure of a population may affect the prevalence of HIV on a number of levels.^25^ ^26^ The prevalence of widowhood, for example, may be an underlying determinant of sex partner acquisition, concurrency, abstinence and sexual mixing patterns.^27^ These are proximate determinants that result in the exposure of susceptible individuals to infected sexual partners. The probability of forming a sexual partnership with an infected individual depends on the prevalence of HIV. We hypothesise that widowhood and other forms of marriage dissolution may have an impact on transmission by supplying a source of infected individuals in the sexual network.

In this paper we describe the demographic patterns of widowhood and the behaviour of widows as they “reconnect” to the sexual network in a setting of high HIV prevalence. The results from these statistical analyses were used to inform the construction of a mathematical model with the aim of estimating the importance of widow behaviour in the heterosexual transmission of HIV.

## METHODS

### Study area, population and survey design

The Manicaland HIV/STD Prevention Study includes a population-based open cohort study, full details of which can be found elsewhere.^28^ The study population are residents of four subsistence farming areas, two roadside trading centres, four forestry, tea and coffee estates and two small towns in the rural province of Manicaland in eastern Zimbabwe. Male and female local residents identified in an initial household census were considered eligible for the study. The baseline round of the study was conducted between July 1998 and January 2000 with the follow-up conducted 3 years later in each site. Men aged 17–54 years and women aged 15–54 years were included in the present analysis. A maximum of one member of each marital group (husband and wife (or wives, in the case of a polygamous union)) was selected to participate in order to maximise the power of an embedded randomised controlled trial.

HIV serological testing was performed on dried blood spots using a highly sensitive and specific antibody dipstick assay.^29^ Information on marital status and marital history in addition to demographic, socioeconomic and sexual behaviour data were collected from each individual through an interviewer-led questionnaire. Responses to sensitive questions about sexual behaviour were collected using an informal confidential system of voting, where participants wrote responses on slips of paper that they inserted into locked boxes. This technique has been shown to improve the discrepancy between male and female reports of sexual activity.^30^

### Statistical methods

#### Prevalence and incidence calculations

For the purposes of prevalence calculations, the baseline and follow-up survey populations were treated as separate cross-sectional studies. Thus, all subjects interviewed in a given study round were included in the denominator of prevalence calculations. Men and women who had not yet started sex were included, but percentages are also presented based only on the sexually experienced population (ie, those who had started to have sex at the time of the interview).

Individuals who were part of a marital union at the time of the baseline survey were considered the population at risk of becoming widowed. The incidence of widowhood was calculated as the number of married people at baseline reporting to be widowed at the time of follow-up divided by the time at risk. The year of the widowing event was available from questionnaire data, but was sometimes missing or inconsistent (eg, a person’s marital status changed to widow in the previous 3 years but they reported that their last partner died >3 years ago). In either situation, we assumed that the person was widowed halfway (1.5 years) through follow-up. Widowing events where a husband and wife in a marital pair both died in the 3-year follow-up period may have been missed if the household dissolved following the deaths, potentially resulting in underestimation of widowing incidence rates. Rates were stratified by gender, age and HIV status at baseline. For the purposes of calculating the incidence of HIV, time-at-risk from all individuals negative at baseline who were found at follow-up were included in the denominator; individuals lost to follow-up were censored halfway through the study period.

oisson regression models were fitted to the widowing incidence data and HIV incidence data and are presented with respect to age, gender and HIV status at baseline (widowing incidence only). Multiple logistic regression was used to model widowhood on HIV status at follow-up. Because age, marital status and HIV prevalence are correlated, we controlled for age in order to describe the independent association of marital status with HIV. Cox proportional hazards models were fitted to remarriage events and are presented with respect to gender, controlling for age. The combination of the questions “How many years is it since your last marital partner passed away?” and “How many years later did you remarry” were used to construct a Kaplan-Meier curve of remarriage. Data on individuals who had been widowed but not yet remarried were included as being censored at the end of the follow-up period.

### Mathematical model of widowhood

Based on the findings from the statistical analysis, a preliminary mathematical model was constructed. While individual risks of infection can be measured directly from cohort studies such as the Manicaland cohort, their contribution to HIV transmission at the population level cannot. The model presented here seeks to examine the importance of male and female widow behaviour in the transmission of HIV, rather than to quantify the impact of interventions targeted at this group. Four sets of alternative assumptions were used to define the baseline pattern of sexual behaviour of widows, where parameters could not be directly based on the survey data. Based on previously described models,^31^ we simulated the heterosexual transmission of HIV in a population stratified by age (5-year categories), sexual activity (three categories with different rates of sexual partnership formation) and marital status (married or not married). Individuals are at risk of becoming widowed if they are currently married, or of getting remarried if they are currently a widow. The risk that a susceptible individual is infected with HIV is dependent on their pattern of sexual partnership formation, which is defined with respect to age (older men with young women) sexual activity (biased to one’s own sexual activity group) and marital status. We investigated the effect of reducing the number of sexual partnerships made by widows under a range of assumptions about the baseline sexual behaviour of widows and non-widows: the rate of partnership formation by widows exceeding that of non-widows; different preferences of widowers and non-widows to form partnerships with younger women; and the extent to which widowers chose widows as sexual partners. The model does not explicitly consider divorced or separated individuals. A full description of the model is presented in the online appendix.

Two parameters were estimated directly from the longitudinal data from the present study: the rates of being widowed, stratified by gender and HIV status, were based on the incidence of widowhood, and the rate of widow remarriage, stratified by gender, was estimated on the basis of the Kaplan-Meier survivor function. Other parameters were selected as described previously.^31^

To illustrate the full contribution of widows to the spread of HIV, we compared a simulation where widows remain active with one where they do not, given different assumptions about their sexual behaviour.

## RESULTS

At baseline there were 4261 men and 5319 women whose marital status was known and at follow-up there were 3341 men and 5096 women resulting from loss to follow-up and new recruitments to the cohort. Among the subjects interviewed and tested for HIV at baseline whose marital status was known, 2242 men and 3265 women were followed up (follow-up rates: 54% for men and 66% for women). Thirteen men and 292 women who were widowed at baseline were identified at follow-up (follow-up rates: 39% for widowers and 80% for widows). An additional 180 men and 219 women with full data from the baseline study were known to have died.

### Incidence and prevalence of widowhood

**Figure 1:**
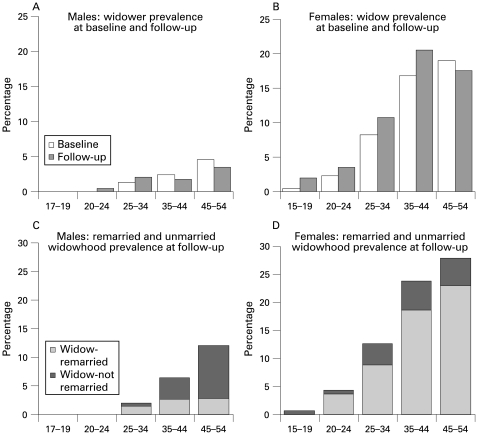
Prevalence of widowhood and remarriage patterns by age and sex in Manicaland, Zimbabwe, 2001–3. (A) Men: prevalence of widowers at baseline and follow-up. (B) Women: prevalence of widows at baseline and follow-up. (C) Men: prevalence of remarried and unmarried widowers at follow-up. (D) Women: prevalence of remarried and unmarried widows at follow-up.

The proportion of sexually experienced men who reported to be widowed was 1.2% at baseline (n = 44) and follow-up (n = 33, table 1). The proportion of sexually experienced women who were widowed increased from 9.4% (n = 410) at baseline to 11.4% (n = 420) at follow-up. Figures 1A and B show that the prevalence of widowhood was higher in men aged <35 years at follow-up. The same trend was observed in women, with the prevalence of widowhood at follow-up being higher up to the age of 45 years.

**Table 1:** Distribution of marital status at baseline (1998/2000) and in follow-up survey (2001/2003)

	Baseline			Follow-up		
	n	% of total	% of ever had sex	n	% of total	% of ever had sex
Men						
Presently widowed	44	1	1	31	1	1
Presently divorced	135	3	4	71	2	3
Presently separated	36	1	1	33	1	1
Presently in union	1734	41	49	1533	47	60
Never married, ever had sex	1602	38	45	874	27	34
Never married, never had sex	710	17		705	22	
Total, ever had sex	3551	83		2542	78	
Total	4261			3247		
						
Women						
Presently widowed	410	8	9	420	9	11
Presently divorced	424	8	10	290	6	8
Presently separated	194	4	4	139	3	4
Presently in union	2968	56	68	2615	54	71
Never married, ever had sex	343	6	8	221	5	6
Never married, never had sex	980	18		1114	23	
Total, ever had sex	4339	82		3685	77	
Total	5319			4799		

Table 2 shows the incidence of widowhood among those who were married at the baseline interview. Nineteen men and 148 women who were married at baseline reported being widowed at follow-up. The incidence rate ratio (RR) with respect to gender (female:male) was 4.1 (95% confidence interval (CI) 2.5 to 6.6, z-test p<0.001, controlling for age). Being infected with HIV at baseline was significantly associated with an increased rate of becoming widowed for women (RR 5.8 (95% CI 4.1 to 8.1), z-test p<0.001) and non-significantly for men (HR 2.3 (95% CI 0.9 to 5.7), z-test p = 0.07), after controlling for age.

**Table 2:** Incidence of widowhood among men (aged 17–49 years) and women (aged 19–49 years) who were married at the time of the baseline interview (1998/2000) until the follow-up interview (2001/2003)

	Married at baseline*	Widowed at follow-up	Years of observation	Incidence per 1000 person-years at risk (95% CI)
Men				
All	1033	19	3070	6.2 (3.9 to 9.7)
HIV-negative	736	10	2192	4.6 (2.5 to 8.7)
HIV-positive	297	9	878	10.2 (5.3 to 19.7)
				
Women				
All	2149	148	6228	23.7 (20.2 to 27.9)
HIV-negative	1738	71	5190	13.9 (11.0 to 17.5)
HIV-positive	411	77	1118	68.9 (55.1 to 86.1)

*Subjects identified at follow-up.

### HIV risk and widowhood

At follow-up the prevalence of HIV was high among ever-widowed men (54%) and women (63%). The prevalence of HIV was higher in widows than in those presently married and in those never widowed for both genders (table 3).

**Table 3:** Prevalence of HIV by widowhood status at follow-up (2001/2003)

	HIV positive/total	Prevalence (%)	OR (95% CI)*
Men			
Presently married, formerly widowed	15/46	54	5.2 (2.7 to 10.0)
Presently widowed	19/31	54	3.9 (1.9 to 8.1)
Presently married, never widowed	403/1465	19	1
Never married, ever had sex	72/868	21	0.7 (0.5 to 0.9)
Never married, never had sex	8/702	1	0.2 (0.1 to 0.5)
			
Women			
Presently married, formerly widowed	70/129	67	5.7 (3.9 to 8.4)
Presently widowed	223/413	61	6.2 (4.9 to 7.9)
Presently married, never widowed	484/2512	28	1
Never married, ever had sex	46/218	8	1.6 (1.1 to 2.3)
Never married, never had sex	16/1105	1	0.2 (0.1 to 0.4)

OR, odds ratio.

*Adjusted for age.

Does not include divorced or separated persons.

For women, the incidence of HIV was not significantly higher in widows (n = 6 seroconversions, 14.7 cases/1000 person-years (pyrs), 95% CI 2.2 to 10.9) than in married women (n = 65 seroconversions, 12.5 cases/1000 pyrs, 95% CI 5.7 to 9.3). Of the 14 men who were widowed and who were HIV-negative at baseline, 2 had died and 7 were lost to follow-up. None of the remaining 5 seroconverted, thus precluding a full analysis of the incidence of HIV in widowed men.

### Mortality

Mortality rates for both male and female widows (95 per 1000 pyrs and 39 per 1000 pyrs) were substantially higher than for other marital status categories (table 4). Mortality rates were higher for widows (RR 3.1, 95% CI 2.1 to 4.4; z-test p<0.001) and widowers (RR 3.7, 95% CI 2.8 to 6.8; z-test p<0.001) than for individuals married at baseline after controlling for unequal age distribution between marital states.

**Table 4:** Mortality rates by marital status at baseline, controlling for age and HIV status

Marital status at baseline	N	Deaths	Rate (per 1000 patient-years)	RR adjusted for age	RR adjusted for age and HIV status
Men					
Presently widowed	30	11	95.2 (52.7 to 172)	3.1 (1.6 to 5.7)	1.8 (1.0 to 3.3)
Presently divorced	105	17	44.8 (27.8 to 72.1)	2.0 (1.2 to 3.4)	1.8 (1.1 to 3.0)
Presently separated	28	1	9.4 (1.3 to 66.7)	0.5 (0.1 to 3.8)	0.5 (0.1 to 3.7)
Presently in union	1374	114	22.7 (18.9 to 27.2)	1	1
Never married, ever had sex	1200	30	6.3 (4.4 to 9.0)	0.8 (0.5 to 1.3)	0.9 (0.6 to 1.5)
Never married, never had sex	587	7	3.3 (1.6 to 6.9)	0.5 (0.2 to 1.4)	0.8 (0.3 to 2.1)
					
Women					
Presently widowed	371	45	38.7 (28.9 to 51.8)	3.1 (2.1 to 4.4)	1.6 (1.1 to 2.3)
Presently divorced	363	35	28.7 (20.6 to 40)	2.6 (1.7 to 3.8)	1.4 (0.9 to 2.0)
Presently separated	168	12	21.3 (12.1 to 37.5)	2.0 (1.1 to 3.6)	1.3 (0.7 to 2.4)
Presently in union	2611	96	11 (9.0 to 13.4)	1	1
Never married, ever had sex	289	19	19 (12.1 to 29.8)	2.2 (1.3 to 3.8)	1.8 (1.1 to 2.9)
Never married, never had sex	867	12	4.1 (2.3 to 7.2)	0.8 (0.4 to 1.7)	1.3 (0.6 to 2.9)

RR, rate ratio.

Mortality rates were higher in HIV-positive widowers than in HIV-negative widowers (62 per 1000 pyrs vs 17 per 1000 pyrs); the same was true for widows (117 per 1000 pyrs vs 51 per 1000 pyrs). Even after controlling for HIV status at baseline, there was increased mortality in widows (RR 2.2, 95% CI 1.2 to 4.1; z-test p = 0.014) and widowers (RR 1.5, 95% CI 1.1 to 2.2; z-test p = 0.03) compared with individuals married at baseline. This suggests that mortality clustering, where one spouse’s death follows their partner’s death, is not completely attributable to HIV in this population.

### Remarriage and sexual behaviour of widows

Seventy-three of 94 widowers (77%) reported sexual activity in the previous year compared with 104 of 297 widows (35%). Traditional practices in Manicaland discourage widows from taking another partner for 1 year after the death of their spouse. Thus, the high levels of reported sexual activity in the first year after widowhood (77% for men, 52% for women) are surprising, but this figure may be influenced by relations with partners who died <1 year ago, as well as recall bias.

**Figure 2:**
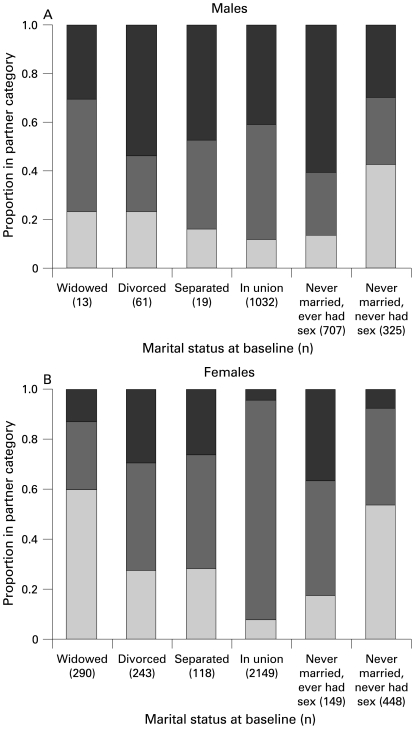
Total number of partnerships in 3-year follow-up period based on marital status at baseline: 0 (light grey), 1 (dark grey), >1 (black).

The majority (modal number) of women widowed at baseline reported no (0) total sexual partnerships in the 3 years of follow-up compared with (1) for women who were separated, divorced, married or unmarried but sexually experienced. However, the mode masks the increased likelihood of extreme levels of sexual activity (⩾10 partners in 3 years) of widows compared with those married at baseline (OR 7.1, 95% CI 2.4 to 20.8; p<0.001, adjusted for age). Only 13 men who were widowed at baseline were found at follow-up, so no comparison could be made. However, separated and divorced men were much more likely to have high sexual activity (⩾10 partners in 3 years) than those married at baseline (OR 4.1, 95% CI 1.4 to 11.6; p = 0.009, adjusted for age). A summary of the partnership history in the follow-up period is shown in fig 2.

Twenty-four of 48 (50%) sexually active widowed women at follow-up reported that they had received money or gifts in exchange for sex with their last partner compared with 83 of 2794 (3%) married women (p<0.001, χ^2^ test).

**Figure 3:**
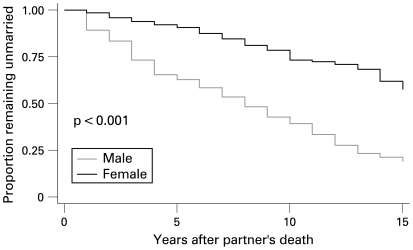
Kaplan-Meier survival plot of remarriage after widowhood (n = 99 men; 644 women).

The hazard ratio of male remarriage to female remarriage was 3.4 (95% CI 2.4 to 4.9, z-test p<0.001 controlling for age; fig 3).

It has previously been shown that the age difference of sexual relations (men being older than their female partners) influences the age patterns of HIV in this population.^28^ There was a greater age difference between widowers and their last partner (median 10.5 years older than partner (interquartile range (IQR) 4.5–15.5)) compared with non-widowers (median 6 years (IQR 3–8); Spearman’s rank test rho = 0.106; p = 0.001). The median age difference between women and their last partner was 6 years (women being younger; IQR 3–10) which was similar between widows and non-widows (Spearman’s rank test rho = −0.0176; p = 0.4).

Thirty-one of 45 widows (69%) reported that their last partner was married to someone else compared with 320/2281 married women (14%; p<0.001, χ^2^ test).

Condom use (either consistent or inconsistent) was more commonly reported by widowed women than by married women (21/40 (53%) vs 151/1921 (8%); p<0.001, χ^2^ test), although HIV-positive widows reported condom use less frequently than HIV-negative widows (10/24 (42%) vs 8/11 (64%); p = 0.11, χ^2^ test).

### Model: dynamic population impacts of widowhood

Parameterisation of the rate of being widowed stratified by gender and HIV status was based on the incidence of widowhood shown in table 2 and the rate of widow remarriage, stratified by gender, was estimated on the basis of the Kaplan-Meier survivor function (1/8 for widowers, 1/15 for widows).

**Figure 4:**
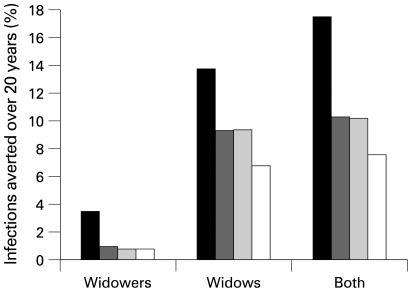
Model prediction. Percentage of cases averted over 20 years after cessation in sexual behaviour of male, female and all widows. Four scenarios of the pre-intervention mixing and sexual activity patterns of widows (W) and non-widows (NW) are presented: (1) Widows form more sexual partners than non-widows, the age difference between partners is higher for widows than non-widows, and widows form most of their partnerships with non-widows (black bars). (2) As for (1), but with the same numbers of sexual partners (dark grey bars). (3) As for (2), but age difference between partners is the same (light grey bars). (4) As for (3), but widows preferentially form partnerships with other widows (white bars).

For the purposes of our baseline model, we assume that the rate of partner change among widows is equal to that of the non-widowed population, with widowed men from older age groups (⩾30 years) having a preference for women 5–10 years younger and widows preferentially selecting non-widows as their partners. Removing widower sexual activity from the model has a minor impact, whereas stopping female sexual activity results in a 2–3% decrease in adult prevalence within 10 years. Figure 4 shows the predicted cases averted (ie, the percentage fewer cases in the intervention compared with the baseline) over a 20-year period in the baseline scenario. These estimates were most sensitive to assumptions about levels of sexual activity of widows and their relationships to the sexual network. If we assume that widowed individuals are four times more likely to form new partnerships, then we estimate that about 17% of infections over 20 years can be attributed directly or indirectly to widow behaviour. If widows form partnerships at the same rate as others, then widow behaviour could underlie approximately 10% of new infections. The estimate is similar if widowers form most of their partnerships with women the same age as themselves (ie, not younger women), but lower (about 8%) if widow(er)s form most of their partnerships with other widow(er)s. This is because more of the partnership of HIV-infected widow(er)s would be widows who are already infected.

## DISCUSSION

This combined statistical analysis and dynamic simulation modelling suggest that widowhood may play an important role in the transmission of HIV in this rural Zimbabwean population. The prevalence of young widowhood, particularly among women, is high (11.4%) in Manicaland. In addition, the prevalence of HIV among widows (63%) and widowers (54%) is exceptionally high and is consistent with the finding that the incidence of becoming widowed is strongly associated with HIV status. However, we found no evidence that widowed men or women had a greater risk of becoming infected with HIV than married individuals, supporting the idea that the high prevalence of HIV among widows is a result of infections acquired while in marital unions rather than as widows. However, widows may be a source of infection as they resume sexual activity, since the sexual relations they form may be very risky for their partners. Men are likely to take on partners much younger than themselves, and women are likely to have relations with already married men and are disproportionately involved in high levels of sexual partner acquisition (⩾10 partners in 3 years) and transactional sex. These relationships may be important means by which HIV is transmitted in a sexual network to the partners of widows and their partners’ partners.

The mathematical model simulations presented here serve to illustrate the relative importance of particular behaviours among widows in the transmission of HIV in this population. The model suggests that, at the population level, the impact of heterosexual transmission of HIV from widows is non-trivial. The cessation of male and widow sexual activity would result in the aversion of 8–17% of cases. Clearly, the cessation of such activity is neither possible nor necessarily desired, but this scenario illustrates the scale against which the impacts of other interventions targeted at widows (such as condom use in commercial relationships or delays in first sex) should be measured. Widow sexual activity is much more important than widower sexual activity in the models, but this prediction is simply explained by the higher observed prevalence of widows in the study population. As female mortality rises as a result of AIDS,^8^ widowers will become more common and therefore their sexual activity would exert a stronger influence. The current model fails to capture this possible future trend. Also not apparent from the population-level perspective of the model is the high risk posed by widowers to their individual partners. They have a very high prevalence of HIV and a preference for women much younger than themselves, meaning that the chances of a susceptible female coming into contact with an infected widower are high. The impact of widow sexual behaviour may be complex, with various aspects of their risk—such as higher levels of condom use and greater age disparity with their partners—having potentially opposing effects. The model may capture some of these dynamics, but not all aspects—including differential levels of condom use by widows and non-widows—were included.

**Take-home messages**

- Widows in Zimbabwe have an exceedingly high prevalence of HIV, mainly because of infection acquired in marriage.
- Many widows resume sexual activity following the death of their partner. Aspects of widow and widower sexual behaviour may put both themselves and their partners at risk.
- Widowhood plays an important role in the transmission of HIV in this rural Zimbabwean population and may be associated with 8–17% of all HIV cases.

The strong sex difference, with widower prevalence at 1.2%, is notable and is likely to be a result of male mortality being generally higher combined with the impact of the early stages of the HIV epidemic which disproportionately affected men, and the pattern of husbands being older than wives.^32^ Similarly, polygamy also affects the sex difference; one male death has the potential to create multiple widows. The 1999 Demographic and Health Survey found 8.4% of men in Manicaland involved in a polygamous union.^22^ In traditional patrilineal family structures, widowed women would be remarried to the brother of their deceased spouse. However, for the inheriting brother, this union would not typically be his first marriage. More often, the inheriting male would be presently married, widowed, separated or divorced. Marriage of widows may become less desirable, particularly in settings where the HIV status is known. In rural Malawi, HIV-positive widows and widowers are already less likely to remarry than HIV-negative widows and widowers.^33^ Remarriage rates for divorcees are higher than those of widows, who are more likely to be perceived as high-risk.^34^ In Uganda, less healthy widows were found frequently to leave their deceased husband’s home and return to their natal village, while healthier widows were more likely to remarry or form new sexual partnerships.^6^

Although participation rates were high, losses to follow-up were substantial. The most common reasons for loss to follow-up in our study were migration out of the study area and households not being found at follow-up.^35^ It is possible that widows may be especially difficult to study since they may move to the town or village of their new spouse or their own extended family. Overall, follow-up of widows was similar to that of the married population. However, it remains possible that people married at baseline who were widowed in the course of follow-up were lost more frequently than those who remained married. In addition, newly widowed men and women may have died before follow-up, causing an underestimation in our estimate of widowhood and widow mortality. Despite efforts to account for all households and their members not found at follow-up, it is possible that households with widows were disproportionately lost. Given their higher mortality rates, households with widows may have been more likely to have been lost to follow-up, particularly if there was a dual death in the course of follow-up.

A limitation of this analysis is that we could only consider patterns of widowhood during a 3-year follow-up period. In order to see if remarriage patterns are changing, a longer study period will need to be analysed, which will be possible as data become available from further rounds. In this analysis we have focused on quantifying contributors to transmission probability and contact patterns with respect to widowhood. However, these individual characteristics and behaviours will depend on local social networks and the cultural milieu, which are predicated on demographic change, legal frameworks and other structural factors.^27^ Although the legal standing of Zimbabwean widows and their rights to inheritance changed in 1997 with the passing of several acts affording them greater protection, widows married under customary law do not have complete security.^36^

In the immediate term, counselling and testing services should reinforce efforts to provide widows with support and knowledge needed to make safe choices after the death of a spouse, and encourage participation in church or women’s organisation support groups where legal advice and support might be sought. In the longer term, if the levirate is declining, increasing financial independence through employment opportunities for widows will reduce their need for support from a new partner. Given the success of the “ABC” approach in helping to lower the prevalence of HIV in Uganda, church and community leaders as well as faith-based organisations should continue to promote safer sexual behaviours such as partner reduction and monogamy within the marital union. In future, as more longitudinal data become available from this population, these behavioural measures should be revisited to determine whether widow practices are changing. The impact of demographic changes on behavioural trends is hard to predict. One interpretation of higher levels of widowhood is that the prospect of remarriage is falling over time,^13^ although this could be part of the natural course of the epidemic. Qualitative research is therefore also needed to provide a more nuanced understanding of changing cultural rules and norms regarding sexual behaviour and marriage patterns in Zimbabwe, and whether these substantiate quantitative results.
